# Adsorption Characterization and Mechanism of a Red Mud–*Lactobacillus plantarum* Composite Biochar for Cd^2+^ and Pb^2+^ Removal

**DOI:** 10.3390/biology15020153

**Published:** 2026-01-15

**Authors:** Guangxu Zhu, Yunhe Zhao, Yunyan Wang, Baohang Huang, Rongkun Chen, Xingyun Zhao, Panpan Wu, Qiang Tu

**Affiliations:** 1 College of Biology and Environment Engineering, Guiyang University, Guiyang 550005, China; 2 Institute of Paleontology, Yunnan University, Kunming 650500, China; zhaoyunhe@stu.ynu.edu.cn; 3 Helmholtz International Lab for Anti-Infectives, Shandong University–Helmholtz Institute of Biotechnology, State Key Laboratory of Microbial Technology, Shandong University, Qingdao 266237, China; 4 Institute of Synthetic Biology Industry, Hunan University of Arts and Science, Changde 415000, China

**Keywords:** distiller’s grain biochar, red mud–*Lactobacillus plantarum* composite, Cd^2+^, Pb^2+^, adsorption mechanism

## Abstract

Pb^2+^ and Cd^2+^ represent common heavy metal contaminants in aquatic environments, posing significant risks to ecosystem stability and human health. To develop efficient adsorbents for removing Cd^2+^ and Pb^2+^ while achieving resource utilization of industrial by-products (red mud and distiller’s grains), this study synthesized a novel composite biochar—red mud–*Lactobacillus plantarum* composite biochar (RM)—by immobilizing red mud and *Lactobacillus plantarum* onto biochar derived from distiller’s grains. The structural and chemical properties of RM were characterized using SEM-EDS, XRD, and FTIR. Batch adsorption experiments were conducted to evaluate the effects of various experimental factors on Cd^2+^ and Pb^2+^ adsorption. The adsorption process was further elucidated through kinetic and isothermal models, revealing that it follows the pseudo-second-order kinetic model. Equilibrium data were best described by the Langmuir model for Cd^2+^ and the Freundlich model for Pb^2+^. The maximum adsorption capacities reached 12.13 mg/g for Cd^2+^ and 130.10 mg/g for Pb^2+^. The primary mechanisms involved in Cd^2+^ and Pb^2+^ adsorption by RM include surface complexation, cation–π interactions, ion exchange, and coprecipitation. These findings demonstrate that RM represents a promising and effective adsorbent for the remediation of heavy metal-contaminated water.

## Simple Summary

Pb^2+^ and Cd^2+^ contamination in water threatens ecosystems and human health. To develop efficient, eco-friendly adsorbents and valorize industrial by-products (red mud, distiller’s grains), this study synthesized a novel ternary composite biochar (RM) by immobilizing red mud and *Lactobacillus plantarum* on distiller’s grain-derived biochar. Characterized via SEM-EDS, XRD, and FTIR, RM exhibited a superior porous structure. Batch adsorption experiments indicated that the adsorption on RM followed pseudo-second-order kinetics, with Cd^2+^ adsorption fitting the Langmuir model and Pb^2+^ fitting the Freundlich model. Key mechanisms included surface complexation, ion exchange, and coprecipitation. RM integrates the synergistic advantages of minerals, microorganisms, and biochar, offering a promising, sustainable solution for heavy metal-contaminated water remediation.

## 1. Introduction

The irregular discharge of industrial and mining wastewater has resulted in severe heavy metal contamination in aquatic environments, representing one of the most critical challenges in modern water management [1]. Among various heavy metal pollutants, lead (Pb) and cadmium (Cd) are of particular concern due to their pronounced toxicity, non-degradability, and tendency to accumulate in biological systems. These characteristics present significant threats to aquatic ecosystems and human health. Both Cd and Pb are recognized for their neurotoxicity and carcinogenic potential, capable of inducing multiple adverse health effects even at low exposure levels [2,3,4]. For instance, lead can damage brain cells and impair cardiovascular, central nervous, endocrine, and digestive functions [5]. Cadmium, on the other hand, may lead to irreversible renal injury, potentially progressing to kidney failure or fatality [6].

Conventional techniques for heavy metal removal include ion exchange, chemical precipitation, electrocoagulation, adsorption, and membrane separation [7,8]. However, the widespread application of these techniques is often hindered by inherent drawbacks: chemical precipitation may generate secondary pollution; ion exchange lacks long-term stability; and membrane separation and electrocoagulation involve high operational costs [9,10]. In comparison, adsorption is considered one of the most promising strategies for remediating heavy metal-laden water, owing to its operational simplicity, ease of control, cost-effectiveness, and high affinity for metal ions [11]. Consequently, a key research direction in adsorption technology involves developing adsorbents that are eco-friendly, economical, and exhibit superior adsorption performance [12].

In recent years, biochar has been widely adopted as a heavy metal adsorbent due to its straightforward preparation and high efficiency, leading to broad applications in water and soil remediation [13,14]. Produced through the pyrolysis of waste biomass under oxygen-limited or anaerobic conditions [15], biochar offers advantages such as abundant raw materials, a porous structure, high specific surface area, and numerous surface functional groups [16,17]. However, the performance of unmodified biochar is highly influenced by pyrolysis temperature and feedstock type, and some variants demonstrate limited effectiveness in practical scenarios [18]. Consequently, increasing attention has been directed toward developing biochar-based composite materials by incorporating other metal-adsorbing substances such as microorganisms, metal oxides, and minerals [19,20,21]. This strategy aims to enhance the physicochemical properties, stability, and adsorption capacity of biochar [22].

Notably, most existing composite adsorbents are confined to binary combinations: typically combining either “biochar + microorganisms” or “biochar + minerals” [23,24,25]. Composites of “biochar + microorganism” utilize microbial metabolic activities for heavy metal transformation or sequestration, supported by the porous structure of biochar [26,27]; however, they often lack the high-density active sites provided by mineral components. Conversely, “biochar + mineral” composites benefit from mineral-driven ion exchange and precipitation [28,29] but fail to utilize microbial capabilities such as microbial biomineralization and extracellular polymeric substance (EPS) secretion. This binary approach leaves a critical research gap: the potential synergistic effects of integrating minerals, microorganisms, and biochar into a ternary composite remain partially unexplored. In such a system, minerals provide abundant active sites, microorganisms could generate precipitation-promoting anions (e.g., CO_3_^2−^, PO_4_^3−^) via metabolism, and biochar could serve as a porous carrier for microbial colonization and ion trapping. To fill this gap, our study aims to experimentally verify whether such synergy exists and contributes to enhanced adsorption performance.

A further limitation lies in the adsorption capacity of existing single or binary adsorbents derived from distiller’s grains or red mud. For instance, unmodified distiller’s grain biochar exhibits a maximum Pb^2+^ adsorption capacity of <80 mg/g [30]. Even in our previous work, distiller’s grain biochar loaded with *Lactobacillus plantarum*—a binary composite, achieved a Cd^2+^ adsorption capacity of only about 9.5 mg/g [31], indicating clear potential for enhancement through the incorporation of mineral components. These limitations underscore the need for a ternary composite that integrates the complementary advantages of minerals, microorganisms, and biochar to achieve superior adsorption performance.

Microbial adsorption offers multiple advantages for heavy metal removal, including high efficiency, low toxicity, cost-effectiveness, and minimal secondary pollution [32]. Numerous studies have demonstrated the capacity of lactic acid bacteria (LAB) to adsorb heavy metals. For instance, Bhakta isolated *Lactobacillus reuteri* Pb71-1 from contaminated sludge and reported a lead adsorption rate of 59% in MRS broth medium [33]. Halttunen et al. found that *B. longum* could adsorb up to 175.7 mg of lead per gram of dry bacterial biomass [34]. In a study by Zhai et al., dietary supplementation with *Lactobacillus plantarum* CCFM8661 in tilapia (*Oreochromis niloticus*) was shown to prevent mortality induced by lead exposure and significantly decrease lead accumulation in the liver, brain, gills, gonads, and muscles of the fish [35]. Our earlier pot experiments further revealed that the application of *Lactobacillus plantarum*, originally isolated from homemade fruit-based garbage enzymes, could markedly lower the bioavailability of heavy metals (lead, cadmium, and zinc) in soil and inhibit their uptake by plants [36].

China is a major producer and consumer of *baijiu* (Chinese liquor). The continuous expansion of the baijiu industry has led to a corresponding increase in the output of distiller’s grains. This by-product is characterized by high moisture content (typically exceeding 70%), high acidity, and residual microorganisms from fermentation, making it prone to rapid decay and foul odor, which significantly limit its direct utilization [37]. However, distiller’s grains are rich in lignocellulosic material, positioning them as a suitable bio-based feedstock for producing biochar adsorbents—materials highly effective in removing toxic contaminants from water. Converting this waste into biochar not only addresses the challenges of its accumulation and disposal but also achieves the resource recovery of an industrial by-product.

Red mud, a solid waste generated during alumina extraction from bauxite, has attracted considerable research interest due to its stable chemical composition, high dispersibility, large specific surface area, strong adsorption capacity, and stability in aqueous environments [38,39]. It readily interacts with and immobilizes metal ions in soil and water, functioning as an effective adsorbent [40]. Furthermore, red mud contains calcium and magnesium ions that can react with soluble carbonates under alkaline conditions to form precipitates, providing additional sites for heavy metal adsorption [41]. Its inherent alkalinity further promotes the immobilization of heavy metal contaminants, particularly in wastewater and contaminated soil [42,43]. Compounds such as Al_2_O_3_, Fe_2_O_3_, CaO, and SiO_2_ present in red mud, especially iron oxides, provide active surface sites that bind heavy metals, thereby reducing their bioavailability [39,44].

Although distiller’s grain biochar, *Lactobacillus plantarum*, and red mud each exhibit promising properties for heavy metal remediation, prior studies -including our own work with binary composites [31] have not combined all three components to harness their complementary advantages: the mineral active sites of red mud, the biomineralization capacity of *Lactobacillus plantarum*, and the porous structural support of biochar. To fill this research gap, this study synthesized a novel ternary composite—red mud–*Lactobacillus plantarum* loaded distiller’s grain biochar (designated as RM)—by immobilizing red mud (mineral component) and *Lactobacillus plantarum* (microorganism) onto distiller’s grain biochar (porous carrier). Batch adsorption experiments were conducted to evaluate the effectiveness of RM in removing Cd^2+^ and Pb^2+^ from aqueous solutions. The physicochemical characteristics of RM before and after adsorption were characterized using scanning electron microscopy with energy dispersive spectroscopy (SEM-EDS), Fourier transform infrared spectroscopy (FTIR), and X-ray diffraction (XRD). The study aims to: (1) demonstrate the superior adsorption performance of the ternary composite, (2) elucidate the adsorption kinetics and isotherms of Cd^2+^ and Pb^2+^ on RM, and (3) investigate the underlying adsorption mechanisms, thereby providing a theoretical foundation for the application of this composite in water pollution remediation.

## 2. Materials and Methods

### 2.1. Biochar Preparation

Distiller’s grains, primarily composed of rice hulls, were sourced from a liquor distillery in Guizhou Province, China. The *Lactobacillus plantarum* strain was isolated from homemade fruit–vegetable garbage enzymes through anaerobic fermentation (35 °C, 36 h) and selectively cultured on MRS medium. This strain exhibits high tolerance to heavy metals and holds potential for bioremediation applications. Red mud was collected from an alumina refinery in Guiyang City, China.

Biochar from distiller’s grains (BC) was prepared via slow pyrolysis under limited oxygen conditions. The distiller’s grains were first dried at 60 °C to constant weight, crushed, and ground. The powdered material was hermetically sealed in a high-temperature-resistant aluminum container covered with a lid and multiple layers of aluminum foil. The sealed container was then placed in a muffle furnace, heated to 450 °C at a rate of 5 °C/min, and maintained at this temperature for 2 h. After pyrolysis, the furnace door was kept closed to allow slow cooling over 8 h until room temperature was reached. The resulting biochar was passed through a 100-mesh sieve to obtain BC.

*Lactobacillus plantarum* suspension was prepared as follows: (1) Strain activation: The preserved strain was inoculated at 1% (*v*/*v*) into 100 mL of sterilized MRS liquid medium and incubated at 35 °C for 24 h. (2) Scale-up culture: The activated culture was transferred at 1% (*v*/*v*) into 1 L of sterilized MRS liquid medium and cultured at 35 °C for 36 h. (3) Cell harvesting: The culture was centrifuged at 3500 rpm for 5 min at 4 °C. The supernatant was discarded, and the pellet was washed twice with 0.85% sterile saline under the same centrifugation conditions. (4) Suspension preparation: The pellet was resuspended in sterile saline to achieve a bacterial concentration with OD600 = 1.0. Aliquots were stored at 4 °C until use.

**Figure 1:**
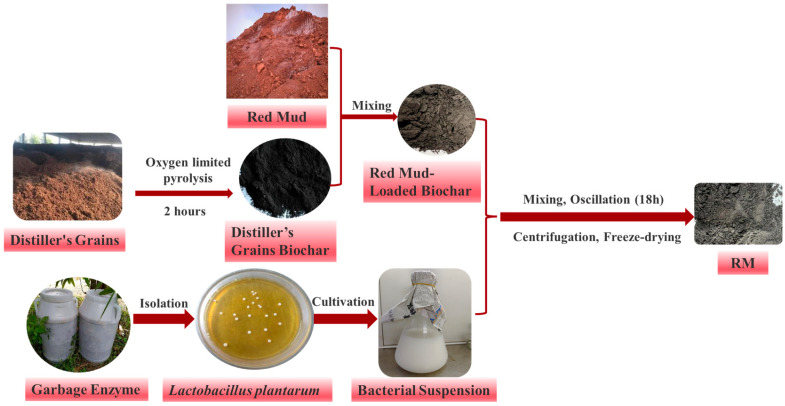
Flow charts of RM preparation.

The red mud-microorganism composite biochar (RM) was synthesized as follows: (1) Matrix preparation: Red mud and distiller’s grain biochar were mixed at a 1:1 mass ratio, ultrasonically cleaned with ultrapure water, dried at 60 °C to constant weight, and sieved through a 100-mesh sieve to obtain a uniform powder mixture. (2) Microorganism loading: The composite matrix was combined with the bacterial suspension at a mass-to-volume ratio of 1:10 (g:mL), and the mixture was shaken at 30 °C and 180 rpm for 18 h. (3) Post-treatment: The culture was centrifuged at 4000 rpm for 5 min to collect the precipitate, which was then washed twice with 0.85% sterile saline via centrifugation. The final product was vacuum freeze-dried to obtain RM (Figure 1).

### 2.2. Characterization of Biochar

Biochar yield is calculated using the gravimetric method [45]. A scanning electron microscope (SEM, ZEISS GeminiSEM 300, Oberkochen, Germany) was used to examine morphological changes in the materials at various magnifications. Elemental composition on the surfaces was performed using an energy-dispersive X-ray spectroscopy (EDS, OXFORD Xplore, Oxford, UK) system. Surface functional groups were characterized via Fourier-transform infrared (FTIR, Thermo Scientific Nicolet iS20, Cupertino, CA, USA) spectroscopy. Crystalline phases were identified using an X-ray powder diffractometer (XRD, Rigaku SmartLab SE, Tokyo, Japan), with diffraction peaks compared to reference patterns in Jade 6.0 software. Specific surface area and porosity were analyzed with an automated surface area and porosity analyzer (ASAP 2460, Irving, CA, USA) via nitrogen adsorption–desorption at 77.3 K. The Barrett–Joyner–Halenda (BJH) method was used to determine pore volume and size distribution, while the Brunauer–Emmett–Teller (BET) method was applied to calculate total specific surface area.

### 2.3. Adsorption Experiment Design

Stock solutions of Cd^2+^ (100 mg/L) and Pb^2+^ (1000 mg/L) were prepared by dissolving cadmium chloride (CdCl_2_) and lead (II) nitrate (Pb(NO_3_)_2_), respectively, in ultrapure water. To maintain constant ionic strength, sodium nitrate (NaNO_3_) was added as a background electrolyte at a concentration of 0.01 mol/L. Working solutions with Cd^2+^ concentrations of 5, 10, 20, 40, 60, and 80 mg/L, and Pb^2+^ concentrations of 50, 100, 200, 400, 600, and 800 mg/L were prepared by diluting the corresponding stock solutions. The pH of each solution was adjusted using 0.1 mol/L sodium hydroxide (NaOH) or 0.1 mol/L nitric acid (HNO_3_).

Batch adsorption experiments were conducted in 50-mL centrifuge tubes containing a predetermined mass of RM and 20 mL of Cd^2+^ or Pb^2+^ solution at a specified concentration. The tubes were sealed and shaken in a thermostatic orbital shaker at 25 °C for predetermined durations. After agitation, the mixtures were centrifuged at 4000 rpm for 5 min, and the supernatants were filtered through 0.22 μm membrane filters.

The adsorption performance of RM toward Cd^2+^ and Pb^2+^ was evaluated by systematically varying the adsorbent dosage, solution pH, initial metal concentration, and contact time, as outlined in Table 1. All experiments, including controls, were conducted in quadruplicate for each condition. Pb^2+^ concentrations in the filtrates were quantified using inductively coupled plasma optical emission spectrometry (ICP-OES, Optima 5300DV, PerkinElmer, Waltham, MA, USA), whereas Cd^2+^ levels were determined via inductively coupled plasma mass spectrometry (ICP-MS, ELAN DRC-e, PerkinElmer, Waltham, MA, USA) due to its higher sensitivity at lower concentration ranges.

The NaNO_3_, HNO_3_, and NaOH used were suprapur reagents, while CdCl_2_, Pb(NO_3_)_2_, and MRS liquid medium were of analytical grade. All these reagents were purchased from Shanghai Aladdin Biochemical Technology Co., Ltd. (Shanghai, China). Ultrapure water was obtained from a Millipore deionization system with a resistivity of 18.2 MΩ·cm.

### 2.4. Data Analysis

#### 2.4.1. Calculation of Adsorption Capacity and Efficiency

The adsorption capacity (q_e_, mg/g) and adsorption efficiency (η, %) were calculated using the following equations [46]:

(1) $$q_{e}=\frac{V(C_{0}-C_{t})}{m}$$

(2) $$\eta =\frac{C_{0}-C_{t}}{C_{0}}\times 100\%$$

where C_0_ and C_t_ are the initial and equilibrium concentrations (mg/L) of Cd^2+^ or Pb^2+^, respectively, V is the solution volume (L), and m is the adsorbent mass (g).

#### 2.4.2. Isothermal Adsorption Model

Adsorption performance of RM was analyzed by fitting data to the Langmuir and Freundlich models [47]:

Langmuir model:

(3) $$\frac{C_{e}}{q_{e}}=\frac{1}{K_{L}q_{m}}+\frac{C_{e}}{q_{m}}$$

Freundlich model:

(4) $$\ln\;q_{e}=\ln\;K_{F}+\frac{1}{n}\ln\;C_{e}$$

where q_e_ is the equilibrium adsorption capacity (mg/g), q_m_ is the maximum adsorption capacity (mg/g), C_e_ is the equilibrium concentration (mg/L), K_L_ is the Langmuir constant, and K_F_ and n are the Freundlich constants.

#### 2.4.3. Kinetic Model

Adsorption kinetics of Cd^2+^ or Pb^2+^ on RM were evaluated using pseudo-first-order and pseudo-second-order models [46]:

Pseudo-first-order:

(5) $$q_{t}=q_{e}(1-e^{-K_{1t}})$$

Pseudo-second-order:

(6) $$q_{t}=\frac{K_{2}q_{e}^{2}t}{1+k_{2}q_{e}t}$$

where qₜ (mg/g) is the adsorption capacity at time t, and K_1_ (min^−1^) and K_2_ (g/(mg·min)) are the rate constants.

## 3. Results

### 3.1. Characterization of RM

#### 3.1.1. Physicochemical Properties of BC and RM

The key physicochemical parameters of the biochars (BC and RM) are summarized in Table 2. BC exhibited an alkaline pH (>7), attributed to the decomposition of biogenic acids during pyrolysis of the rice husk-based distiller’s grains and the presence of inherent alkaline inorganic components [48]. In contrast, RM displayed a lower pH. Although raw red mud is alkaline and would typically raise pH, extensive washing during microbial immobilization and the generation of organic acids by *Lactobacillus plantarum* collectively led to a more neutral final pH. By utilizing pre-formed BC as a base, the preparation of RM achieves a higher biochar yield.

BET analysis indicated that RM possesses a specific surface area 1.65 times larger and a mesopore volume 1.40 times greater than those of BC. The average pore size also decreased from 15.8109 nm to 13.7793 nm. These changes likely result from the incorporation of red mud particles and microbial cells, which partially occupy pore spaces within the biochar matrix. While the absolute BET surface area of RM (8.7773 m^2^/g) is lower than that of many functionalized biochars, this structural optimization still enhances ion trapping and shortens diffusion paths, providing auxiliary support for adsorption. However, the superior adsorption performance of RM is predominantly driven by chemical interactions (e.g., ion exchange, surface complexation, coprecipitation) rather than textural properties alone [49]. Generally, a larger surface area and higher pore volume can enhance a material’s adsorption capability by providing more active sites [49], but in RM’s case, these textural improvements work synergistically with chemical interactions to achieve optimal performance.

#### 3.1.2. Surface Morphology and Elemental Composition of RM

**Figure 2:**
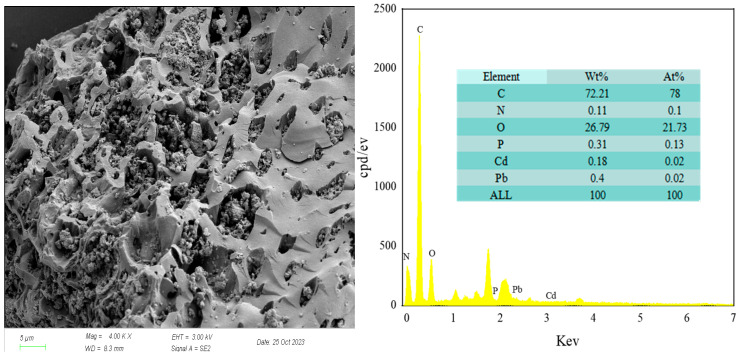
SEM and EDS spectra of RM.

SEM images (Figure 2) revealed a complex and porous network on the RM surface. High-temperature pyrolysis disrupts the original biomass structure, and the release of volatiles generates a multitude of pores [50]. Additionally, short rod-shaped cells of *Lactobacillus plantarum* were observed adhering to the pore walls. EDS analysis identified that carbon was the predominant element (72.21 wt%), accompanied by oxygen, nitrogen, and phosphorus. These elements primarily originate from cellulose, which transforms into an aromatic carbon skeleton during thermal decomposition [51].

#### 3.1.3. FTIR Analysis of RM

**Figure 3:**
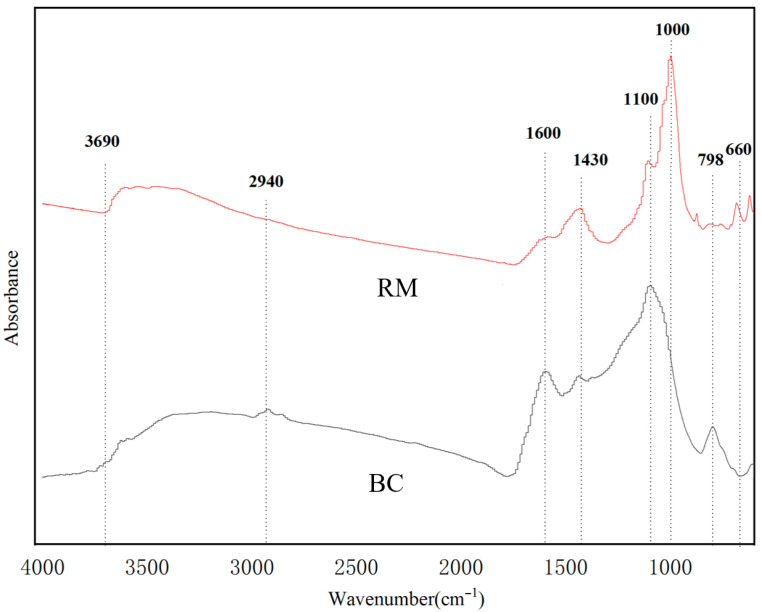
FTIR spectra of BC and RM.

The FTIR spectra of BC and RM (Figure 3) were broadly similar but exhibited distinct band shifts. An intensified absorption near 3690 cm^−1^ corresponds to O–H stretching, likely due to hydrogen bonding in carbohydrates such as cellulose and hemicellulose [52]. The aliphatic C–H stretching band around 2940 cm^−1^ was noticeably reduced, whereas a significant enhancement was observed for the carbonyl C=O band near 1600 cm^−1^. Changes in the region around 1440 cm^−1^ are related to C–H bending in alkyl groups. The variation near 1110 cm^−1^ is assigned to C–O stretching in glycosidic structures [53]. The shift around 1000 cm^−1^ may be associated with SO_3_^2−^ vibrations. Alterations in the 660–796 cm^−1^ region, attributed to Si–O–Si vibrations, suggest a reduction in the polarity and aromaticity of the biochar [54].

#### 3.1.4. XRD Analysis of RM

**Figure 4:**
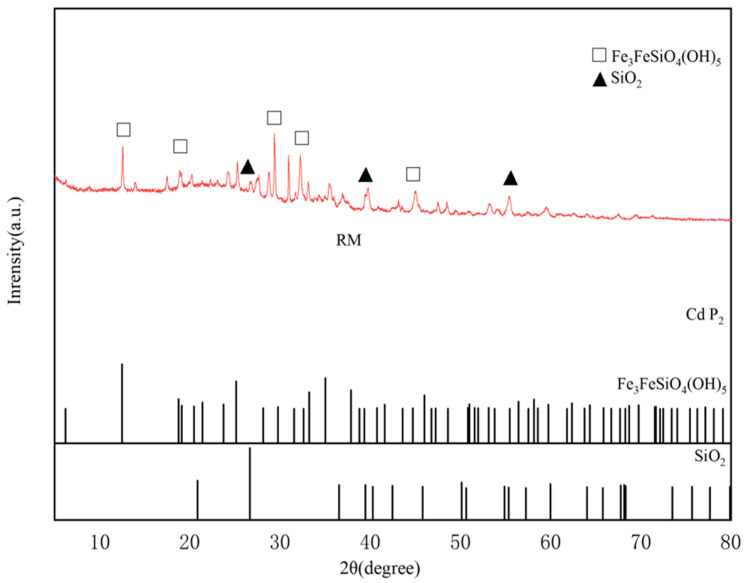
XRD pattern of RM.

The XRD pattern of RM (Figure 4) revealed the presence of crystalline phases containing iron and silicon. Peaks at 2θ = 12.54°, 29.38°, and 32.22° were identified as Fe_3_FeSiO_4_(OH)_5_, with the strongest diffraction peak observed at 29.38°. Additional reflections at 2θ = 26.76°, 39.76°, and 55.52° were assigned to a SiO_2_ phase [55].

### 3.2. Adsorption Performance of RM for Cd^2+^ and Pb^2+^

#### 3.2.1. Effect of Adsorbent Dosage

**Figure 5:**
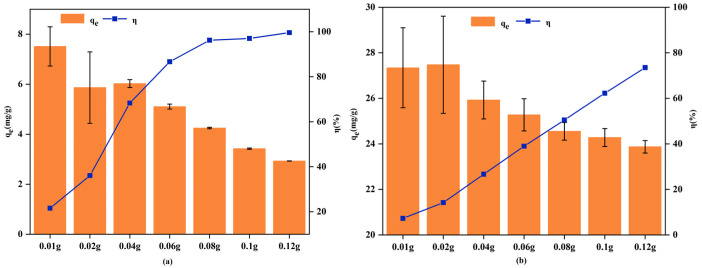
Effect of RM dosage on the adsorption of Cd^2+^ (**a**) and Pb^2+^ (**b**).

The influence of RM dosage on the adsorption of Cd^2+^ and Pb^2+^ is shown in Figure 5. For both metals, increasing the adsorbent dosage enhanced the removal efficiency but decreased the adsorption capacity per unit mass.

When the RM dosage was increased from 0.01 g to 0.08 g, the removal efficiency for Cd^2+^ and Pb^2+^ rose sharply from 21.58% and 7.27% to 96.24% and 50.49%, respectively. Concurrently, the adsorption capacity decreased from 7.52 mg/g to 4.25 mg/g for Cd^2+^, and from 27.34 mg/g to 24.55 mg/g for Pb^2+^. A further increase in dosage to 0.12 g resulted in near-complete removal of Cd^2+^ (99.58%) and a Pb^2+^ removal of 73.50%, suggesting that higher dosages could further improve Pb^2+^ adsorption. The decrease in adsorption capacity with increasing dosage may be associated with particle aggregation and overlapping or interference of adsorption sites at higher adsorbent loadings [56].

#### 3.2.2. Effect of Solution pH

**Figure 6:**
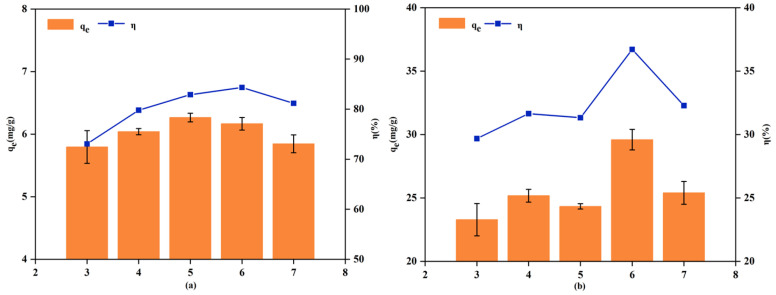
Effect of pH on the adsorption of Cd^2+^ (**a**) and Pb^2+^ (**b**).

The influence of initial solution pH on the adsorption of Pb^2+^ and Cd^2+^ by RM is presented in Figure 6. Experiments were conducted in the pH range of 3.0–7.0 to prevent metal precipitation at higher pH values. At pH 3.0, both adsorption capacity and removal efficiency were low, which is attributed to the competitive adsorption between H^+^ ions and metal cations for the available surface sites. As the pH increased, both adsorption capacity and removal efficiency improved gradually. This enhancement is ascribed to the decreasing concentration of H^+^ ions, which reduces competitive inhibition and thereby makes more adsorption sites accessible for Cd^2+^ and Pb^2+^ binding [57]. The optimal removal efficiencies for Cd^2+^ (84.33%) and Pb^2+^ (36.71%) were achieved at pH 6.0, which was consequently selected for all subsequent adsorption tests.

#### 3.2.3. Adsorption Kinetics

**Figure 7:**
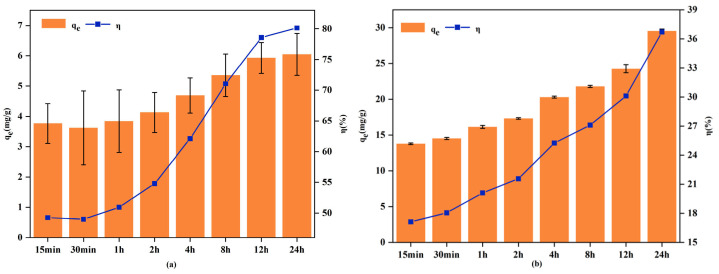
Effect of reaction time on the adsorption of Cd^2+^ (**a**) and Pb^2+^ (**b**).

Figure 7 depicts the influence of contact time on the adsorption of Cd^2+^ and Pb^2+^ from aqueous solution by RM. The results demonstrate that both adsorption capacity and removal efficiency increased rapidly within the initial 12 h, with a subsequent gradual slowdown between 12 and 24 h. This trend can be explained by an initial stage of rapid adsorption, during which abundant active sites were accessible for heavy metal uptake, facilitating the swift migration of ions to the adsorbent surface. As adsorption progressed, the concentration of metal ions in the solution gradually declined, resulting in a diminished concentration gradient. Concurrently, the active sites on RM became increasingly occupied by heavy metal ions, leading to a slower adsorption rate in the later stage until equilibrium was ultimately achieved.

**Figure 8:**
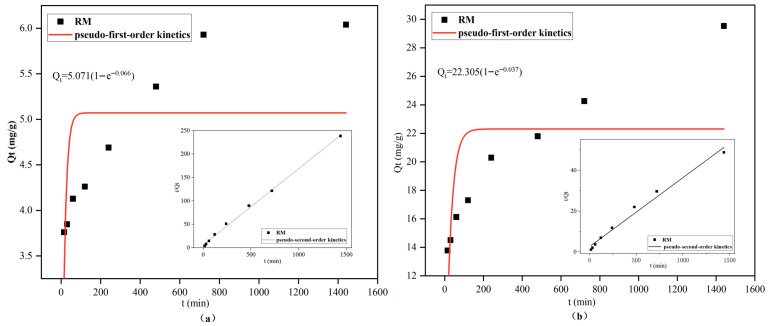
Kinetic model fitting curves for Cd^2+^ (**a**) and Pb^2+^ (**b**) adsorption by RM.

To elucidate the adsorption mechanisms of Cd^2+^ and Pb^2+^ onto RM, the experimental data were fitted with pseudo-first-order and pseudo-second-order kinetic models. The fitting parameters are summarized in Table 3. For both Cd^2+^ and Pb^2+^, the pseudo-second-order model yielded higher correlation coefficients (R^2^) than the pseudo-first-order model, indicating a more accurate description of the adsorption process. Moreover, the theoretical equilibrium adsorption capacities calculated using the pseudo-second-order model were in closer alignment with the experimental values (Figure 8), further confirming the suitability of this model for characterizing the adsorption of Cd^2+^ and Pb^2+^ onto RM. Collectively, these results suggest that the removal of both metal ions is dominated primarily by chemical adsorption, involving processes such as complexation with oxygen-containing functional moieties, coprecipitation, and cation–π interactions [58].

Notably, the pseudo-second-order rate constant (k_2_) of RM for Cd^2+^ was 0.0041 g/(mg·min), which is significantly higher than that of the binary distiller’s grain biochar–*Lactobacillus plantarum* composite (0.0025 g/(mg·min)) reported previously [31]. This enhanced adsorption rate can be attributed to the synergistic effect of the ternary composite: red mud provides abundant active sites to accelerate ion binding, *Lactobacillus plantarum* secretes extracellular polymeric substances that facilitate surface complexation, and the porous structure of biochar shortens ion diffusion pathways—collectively promoting rapid chemical adsorption.

#### 3.2.4. Adsorption Isotherms

**Figure 9:**
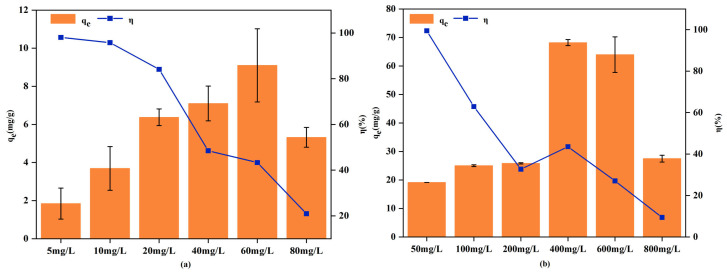
Effect of initial concentration on the adsorption of Cd^2+^ (**a**) and Pb^2+^ (**b**) by RM.

Figure 9 illustrates the effect of initial concentration on the adsorption of Cd^2+^ (a) and Pb^2+^ (b) by RM. As shown, the adsorption capacity of RM for both metal ions initially increased and then decreased with risinginitial concentrations, while the removal efficiency steadily declined. When the initial concentrations of Cd^2+^ and Pb^2+^ increased from 5 mg/L and 50 mg/L to 80 mg/L and 800 mg/L, respectively, the corresponding removal efficiencies dropped from 98.07% to 20.95% for Cd^2+^ and from 99.47% to 9.44% for Pb^2+^. This trend can be explained by the limited availability of adsorption sites. At low concentrations, abundant active sites on RM allow efficient binding of metal ions, enabling rapid uptake without saturation. However, as the number of metal ions increases, the limited active sites and functional groups become saturated, resulting in a decrease in removal efficiency.

In contrast to the findings reported by Kołodyńska et al. [59] and Komkiene and Baltrenaite [60], where biochar adsorption capacity increased continuously with metal concentration, the present study identified that maximum adsorption capacities for Cd^2+^ and Pb^2+^ occurred at 60 mg/L and 400 mg/L, respectively. This discrepancy is primarily attributed to two aspects: first, the active sites on the RM surface tend to saturate as metal ion concentration rises; second, high heavy metal concentrations can induce lipid peroxidation in cell membranes [61], leading to a sharp reduction in the secretion of extracellular polymeric substances by immobilized *Lactobacillus plantarum* and a significant weakening of biomineralization. The simultaneous decline in physical adsorption and biological fixation thus contributes to the decrease in adsorption capacity.

The isothermal adsorption characteristics of adsorbents are commonly described using the Langmuir and Freundlich models. The Langmuir model is a theoretically derived equation based on the assumption that the adsorbent surface contains a finite number of identical active sites. Adsorption reaches a maximum when all sites are occupied, corresponding to ideal monolayer coverage on a homogeneous surface without interaction between adsorbed molecules. In contrast, the Freundlich model is an empirical equation that does not approach a maximum adsorption capacity. It is widely applied to describe multilayer adsorption on heterogeneous surfaces, where the adsorbed amount increases with adsorbate concentration [62].

**Figure 10:**
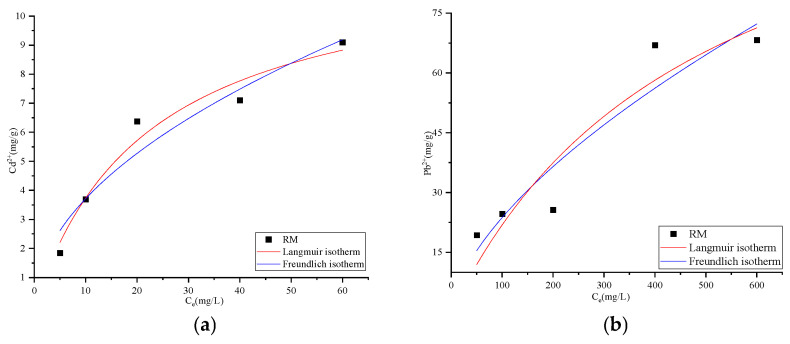
Langmuir and Freundlich isotherm fitting curves for Cd^2+^ (**a**) and Pb^2+^ (**b**).

Figure 10 presents the adsorption isotherms of Cd^2+^ and Pb^2+^ on RM, which show that the adsorption capacities of both metal ions increased with the equilibrium concentration. The experimental data were fitted to the Langmuir and Freundlich models, with the corresponding parameters summarized in Table 4. Based on the comparison of determination coefficients (R^2^), the Langmuir model yielded a better fit for Cd^2+^ adsorption (R^2^ = 0.9557) than the Freundlich model (R^2^ = 0.9203), suggesting that the adsorption of Cd^2+^ likely occurs as a monolayer on a homogeneous surface with uniform active sites [63]. The maximum adsorption capacity (qₘ) obtained in this study was 12.13 mg/g for Cd^2+^, which is higher than the values reported by He et al. using biochar-immobilized cadmium-resistant bacterial consortia (~10.2 mg/g, [64]) and also exceeds the capacity observed in our earlier study on distiller’s grain biochar loaded with *Lactobacillus plantarum* (~9.5 mg/g, [31]). These results demonstrate that RM exhibits superior adsorption potential for Cd^2+^ compared to the binary composites mentioned.

For Pb^2+^, the Freundlich model yielded a better fitting (R^2^ = 0.8511) than the Langmuir model (R^2^ = 0.8390), indicating that the adsorption behavior is consistent with the general trend of multilayer adsorption on heterogeneous surfaces [65], though the moderate fitting quality (R^2^ < 0.86) suggests deviations from ideal multilayer adsorption assumptions. This moderate fitting is attributed to RM’s structural and mechanistic complexity: the ternary composite’s heterogeneous surface (composed of biochar pores, red mud particles, and microbial cells) and the coexistence of multiple adsorption mechanisms (ion exchange, surface complexation, coprecipitation) result in non-ideal adsorption behavior that cannot be fully described by classical isotherm models [21,65].

The difference in adsorption behavior between Cd^2+^ and Pb^2+^ can be attributed to their inherent ionic properties: Pb^2+^ has a larger ionic radius (119 pm) and lower charge density (18.4 C/m^3^), whereas Cd^2+^ has a smaller ionic radius (97 pm) and a higher charge density (24.7 C/m^3^) [66]. These properties reduce Pb^2+^’s affinity for individual active sites, favoring its accumulation on heterogeneous surfaces in a non-ideal multilayer manner—consistent with the Freundlich model’s tendency to describe adsorption on heterogeneous media [61]. The 1/n values from the Freundlich model were below 1 for both metals (0.5051 for Cd^2+^, 0.6218 for Pb^2+^), implying favorable adsorption governed in part by chemical interactions [67], which is supported by kinetic and characterization data (pseudo-second-order model fit, XRD-detected precipitates).

### 3.3. Characterization After Adsorption

#### 3.3.1. Surface Morphology and Elemental Composition

**Figure 11:**
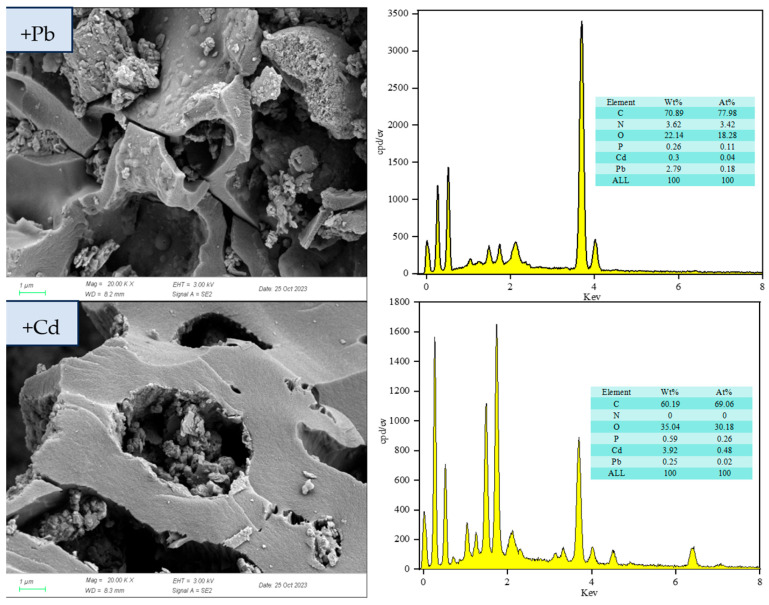
SEM + EDS analysis of RM after Cd^2+^ and Pb^2+^ adsorption.

The evolution in surface morphology and elemental distribution of the composite material after the adsorption of Cd^2+^ and Pb^2+^ was systematically characterized using SEM-EDS (Figure 11). Relative to the pristine sample, the metal-laden samples exhibited notable surface alterations: the lead-loaded sample (RM-Pb) developed a honeycomb-like, wrinkled morphology, while the cadmium-loaded sample (RM-Cd) displayed a corrugated and pitted surface topography. Both samples exhibited micron-scale particle deposits, indicating distinct interfacial reactions at the solid–liquid interface. X-ray elemental mappings revealed that these deposits were enriched in biologically relevant elements such as S and P, indicating that the composite precipitates were likely generated through biomineralization involving microbial metabolites and heavy metal ions. This synergistic mechanism aligns with findings from previous studies [68]. Furthermore, the pores of RM appeared collapsed and deformed after metal adsorption, resulting in a rougher surface texture. The surface of RM-Pb also exhibited raised, blister-like structures. EDS analysis confirmed the presence of Pb and Cd both on the surface and within the material: in RM–Cd, Cd accounted for 3.92 wt%, with Pb at 0.25 wt%; in RM–Pb, Cd constituted 0.30 wt%, while Pb reached 2.79 wt%.

#### 3.3.2. FTIR Analysis

**Figure 12:**
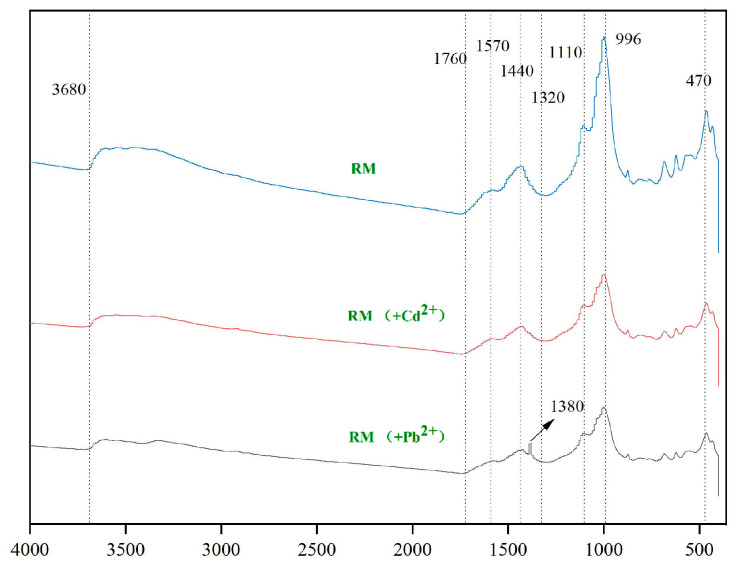
FTIR spectra of RM after Cd^2+^and Pb^2+^ adsorption.

**Figure 13:**
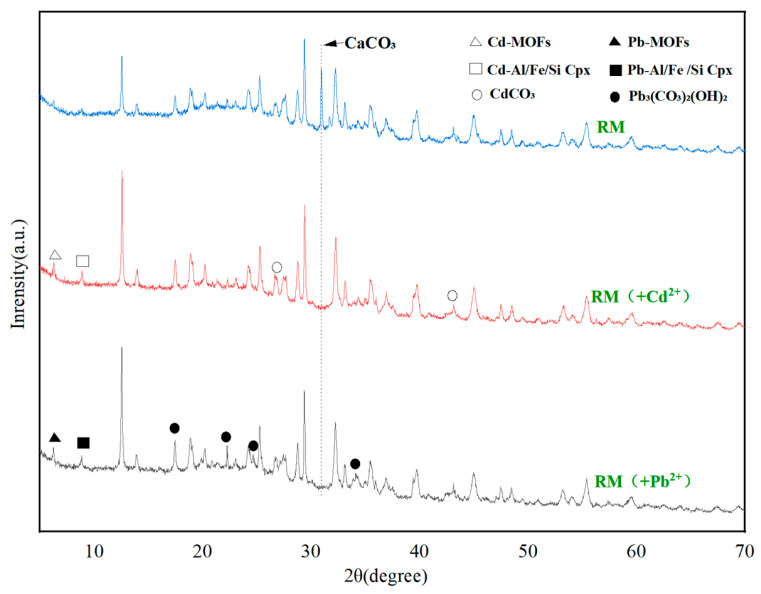
XRD patterns of RM after Cd^2+^and Pb^2+^ adsorption.

FTIR spectra of RM before and after metal adsorption (360–4000 cm^−1^) are presented in Figure 12. Following Cd^2+^ adsorption, no appreciable alterations in functional groups were detected. This observation is attributed to the dominance of strong, signal-intensive mechanisms (coprecipitation and ion exchange) that mask weak interactions such as surface complexation—a phenomenon well-documented in composite adsorbents with multiple adsorption pathways [53,67]. Specifically, XRD (Figure 13) confirms the formation of CdCO_3_ precipitates, and the XRD peak disappearance of CaCO_3_ (2θ = 30.91°) verifies ion exchange between Cd^2+^ and Ca^2+^ from red mud. These two dominant processes generate strong chemical signals that overshadow the weak FTIR responses from Cd^2+^ complexation with O-H (around 3690 cm^−1^, attributed to carbohydrates and red mud minerals) and C=O groups (near 1600 cm^−1^, from biochar carboxyl groups) on RM. While direct FTIR evidence for surface complexation is lacking, this interaction is indirectly supported by: (i) the Langmuir isotherm fit (R^2^ = 0.9557, Table 4), which implies monolayer adsorption on homogeneous active sites (consistent with specific functional group binding) [62]; and (ii) literature reports that *Lactobacillus plantarum*-derived EPS and biochar oxygen-containing groups readily form weak complexes with Cd^2+^ [31,33].

Similarly, the FTIR spectra of RM before and after Pb^2+^ adsorption exhibited no major shifts in the positions of the primary functional group peaks. However, a new stretching vibration peak emerged in the region of 1380–1390 cm^−1^, which is characteristic of phenolic hydroxyl groups [69]. This result provides direct evidence for complexation between Pb^2+^ and phenolic hydroxyls on RM—consistent with the higher affinity of Pb^2+^ for aromatic functional groups (supporting cation–π interactions) compared to Cd^2+^ [66]. For Pb^2+^, the combination of this new peak and XRD-detected Pb(CO_3_)_2_(OH)_2_ precipitates confirms that both surface complexation (phenolic hydroxyl binding) and coprecipitation contribute to adsorption.

#### 3.3.3. XRD Analysis

Figure 13 presents the XRD patterns of RM following the adsorption of Cd^2+^ and Pb^2+^. After the adsorption of Cd^2+^, the disappearance of the diffraction peak at 2θ = 30.91°, previously indexed to CaCO_3_, indicates that ion exchange occurred between RM and Cd^2+^/Pb^2+^ during the adsorption process [70]. Simultaneously, the emergence of new diffraction peaks at 2θ = 6.28° and 8.89° may be tentatively attributed to potential coordination polymers (tentatively denoted as Cd/Pb-MOF-like structures) [71]. These peaks are consistent with the low-angle diffraction features of metal–organic frameworks (MOFs) or coordination polymers formed through interactions between organic ligands (e.g., EPS-derived carboxyl, hydroxyl groups from *Lactobacillus plantarum*, and biochar functional groups) and Cd/Pb ions [72]. However, we acknowledge that these peaks may overlap with other low-crystallinity phases (e.g., metal-hydroxide-organic hybrids), so this assignment is tentative. Additional peaks at 2θ = 26.67° and 43.16° correspond to the formation of CdCO_3_ [73], a definitive phase with well-matched reference patterns.

In the case of Pb^2+^ adsorption, the observed peaks at 2θ = 17.45°, 22.26°, 24.63°, and 34.22° are definitively assigned to Pb(CO_3_)_2_(OH)_2_ [74] (reference pattern: JCPDS 01-074-1824). The weak, broad peaks at 2θ = 6.51° and 9.12° are similarly tentatively associated with Pb-MOF-like coordination polymers. Again, overlap with other amorphous phases cannot be excluded, and further verification is required.

## 4. Discussion

### 4.1. Mechanistic Analysis of Cd^2+^ and Pb^2+^ Adsorption by RM

The adsorption of Pb^2+^ and Cd^2+^ onto RM involves a synergistic effect of textural optimization and dominant chemical interactions. The enhanced adsorption performance is primarily attributed to chemical interactions, with textural improvements playing an auxiliary role. BET analysis revealed that RM possesses a specific surface area 1.65-fold larger and a mesopore volume 1.40-fold greater than those of BC, and a reduced average pore size (13.7793 nm). This structural optimization enhances ion trapping in micro–mesoporous networks and shortens diffusion paths, facilitating the initial rapid adsorption stage. However, the core drivers of high adsorption capacity are chemical interactions, including: (i) interfacial complexation mediated by mineral phases (red mud-derived Fe_2_O_3_, CaO, Al_2_O_3_) and oxygen-containing functional groups (biochar’s C=O, O–H; microbial EPS); (ii) ion exchange between heavy metal ions and Ca^2+^/Mg^2+^ from red mud; and (iii) hypothesized precipitation reactions driven by anions (e.g., CO_3_^2−^ and PO_4_^3−^) inferred to be produced via microbial metabolism [35,68]. Notably, the pore size distribution analysis showed a reduction in the average pore size from 15.8409 nm to 13.7793 nm, which may further boost ion-exchange efficiency by improving pore-filling effects. Compared with our previous binary composite (*Lactobacillus plantarum*-immobilized distiller’s grain biochar) for Cd^2+^ adsorption [31] and Shi’s red mud-loaded distiller’s grain biochar for Pb^2+^ adsorption [75], RM exhibits significantly higher adsorption capacity and removal efficiency for Cd^2+^ under all tested experimental conditions, while also outperforming the latter in Pb^2+^ adsorption.

Regarding the functional form of *Lactobacillus plantarum* in RM, a comprehensive inference based on existing characterization and literature supports a synergistic effect of metabolically active cells, inactive biomass, and cell wall functional groups. First, the formation of CdCO_3_ and Pb(CO_3_)_2_(OH)_2_ precipitates detected by XRD (Figure 13) provides indirect evidence of metabolically active cells: *Lactobacillus plantarum* produces organic acids and CO_3_^2−^ via glycolysis [35], which react with heavy metal ions to form precipitates; this process is uniquely dependent on metabolic activity. Second, FTIR analysis (Figure 3) shows enhanced intensity of the C=O band (1600 cm^−1^) in RM compared to BC, which aligns with the binding of heavy metals to carboxyl, amino, and hydroxyl groups on the bacterial cell wall [33]. This surface complexation is independent of metabolic activity and can be achieved by inactive biomass or cell wall fragments. Third, our previous pot experiment confirmed that this strain, when immobilized on biochar, significantly reduces the bioavailability of Cd, Pb, and Zn in soil [36], an effect attributed to both EPS secretion (active metabolism) and cell wall adsorption (structural function). Thus, the contribution of *Lactobacillus plantarum* to RM’s adsorption performance is multifaceted: biomineralization (CO_3_^2−^ and EPS production) relies on metabolically active cells, while surface complexation can be accomplished by inactive biomass or cell wall functional groups; both pathways are supported by the characterization data in this study.

Integrated characterization using SEM-EDS, FTIR, and XRD revealed the formation of particulate deposits on the adsorbent surface following metal uptake. For the Pb-loaded sample (RM-Pb), FTIR analysis showed an enhanced intensity of functional group-related bands, while XRD identified newly formed crystalline phases of Pb-MOFs, Pb–Si–Fe/Al complexes and Pb(CO_3_)_2_(OH)_2_, confirming the incorporation of Pb^2+^ from the solution. These observations suggest that lead adsorption proceeds mainly through ion exchange and surface precipitation.

In the case of the Cd-loaded system (RM-Cd), the FTIR spectra displayed no new absorption bands or notable shifts in key functional groups (e.g., O–H around 3690 cm^−1^ and carboxyl near 1600 cm^−1^), which is attributed to the relatively weak complexation of Cd^2+^ with these groups, which is overshadowed by other dominant mechanisms such as pore confinement, precipitation, and ion exchange. In contrast, XRD analysis revealed distinct diffraction peaks: definitive peaks at 2θ = 26.67° and 43.16° corresponding to CdCO_3_ [72], and tentative broad peaks at 2θ = 6.28° and 8.89° tentatively assigned to Cd-MOF-like coordination polymers (formed via organic ligand-Cd^2+^ interactions) [70]. EDS results further confirmed the increased presence of Cd on the material. These collective findings strongly suggest that cadmium immobilization is governed predominantly by definitive mechanisms (precipitation as CdCO_3_, ion exchange, and surface complexation) with tentative contributions from coordination polymers—this view is consistent with the high correlation coefficients obtained from the Langmuir isotherm and pseudo-second-order kinetic models. The tentative assignment of MOF-like structures requires future confirmation via complementary techniques (e.g., XPS for metal-ligand bonding, TEM for structural morphology, or Raman spectroscopy for organic-inorganic coordination).

### 4.2. Implications for Application and Future Perspectives

While the regeneration of red mud–*Lactobacillus plantarum* composite biochar (RM) will be explored in future studies, the management of the spent adsorbent is critical for its practical application. Several end-of-life strategies can be considered to ensure environmental safety and resource efficiency. For environmentally responsible disposal, the low leachability of adsorbed Cd^2+^ and Pb^2+^—evidenced by XRD identification of insoluble crystalline phases such as CdCO_3_, and Pb(CO_3_)_2_(OH)_2_—combined with the innate stability provided by the red mud matrix, justifies the application of solidification/stabilization (S/S) treatment. Incorporating the spent adsorbent into cementitious or clay-based binders can further immobilize any residual metals prior to final disposal, with leaching tests (e.g., TCLP) confirming compliance with regulatory standards.

Regarding potential recyclability, mild acid elution (e.g., 0.1–0.5 M HNO_3_ or HCl) can effectively desorb metals by dissolving surface precipitates and complexes. Subsequent neutralization and reinoculation with *Lactobacillus plantarum* can restore the material’s adsorption capacity. An alternative, more sustainable regeneration route could employ microbially facilitated desorption using bio-secreted chelators. From a resource recovery perspective, thermal treatment at 600–800 °C under an inert atmosphere allows for the volatilization and subsequent recovery of Cd and Pb. Meanwhile, the resulting mineral-rich ash—containing red mud-derived Al_2_O_3_, Fe_2_O_3_, and SiO_2_—offers potential for valorization in construction or ceramic industries, consistent with circular economy principles. Collectively, these strategies highlight the environmental adaptability of RM and reinforce its potential for sustainable application in heavy metal remediation.

To fully evaluate RM’s long-term applicability and economic feasibility, future research will systematically investigate its regeneration, reuse, and stability: (1) Optimize desorption and regeneration by screening eco-friendly desorbents (0.1–0.5 M HNO_3_, HCl, citric acid, or EPS-derived microbial chelators) and adjusting eluent concentration, contact time, and temperature to achieve >80% metal recovery while preserving active sites; (2) Assess reusability via 3–5 consecutive adsorption–desorption cycles, monitoring adsorption capacity retention (>70% after 5 cycles), removal efficiency, and structural integrity (SEM-EDS, FTIR), with parallel microbial reinoculation experiments to test enhanced recyclability; (3) Characterize stability by evaluating structural changes (BET, pore volume, XRD), conducting leaching tests (TCLP, SPLP) for minimal ion release, and assessing adsorption performance after 1–3 months of dry room-temperature storage.

To enhance the environmental relevance and practical applicability of RM, future research will focus on three key directions targeting Cd^2+^ and Pb^2+^ co-contamination scenarios: (1) Binary competitive adsorption experiments to investigate mutual interference between Cd^2+^ and Pb^2+^, quantify selective adsorption coefficients, and clarify whether RM exhibits preferential binding to either metal under co-occurrence conditions; (2) Evaluation of common coexisting ions (e.g., Na^+^, Ca^2+^, Mg^2+^, SO_4_^2−^, NO_3_^−^) at concentrations typical of industrial wastewater (0.01–0.1 mol/L) to assess RM’s tolerance and adsorption stability; (3) Adsorption tests using actual water matrices (e.g., electroplating wastewater, contaminated surface water) to verify RM’s performance under complex conditions, including the influence of dissolved organic matter and suspended solids, and optimize process parameters (e.g., dosage, pH adjustment) for real-world deployment.

## 5. Conclusions

(1) This study innovatively develops a ternary composite biochar (RM) by immobilizing red mud (mineral component), *Lactobacillus plantarum* (microorganism), and distiller’s grain-derived biochar (porous carrier), filling the research gap of underutilized synergies between three types of components in existing binary composite adsorbents. Compared with binary counterparts (e.g., biochar + *Lactobacillus plantarum*, biochar + red mud), RM exhibits enhanced adsorption capacities for Cd^2+^ (12.13 mg/g) and Pb^2+^ (130.10 mg/g), verifying the superior performance of the ternary integration strategy.
(2) The adsorption of Cd^2+^ and Pb^2+^ by RM is governed by synergistic chemical interactions (surface complexation, ion exchange, coprecipitation) with auxiliary textural optimization. Distinct adsorption behaviors (Langmuir monolayer for Cd^2+^, Freundlich multilayer for Pb^2+^) arise from the interplay between metal ionic properties and RM’s heterogeneous surface, providing new insights into the design of composite adsorbents for targeted heavy metal removal.
(3) RM demonstrates promising practical potential for heavy metal-contaminated water remediation, supported by feasible end-of-life management strategies (solidification/stabilization, regeneration, resource recovery). This study lays a theoretical and technical foundation for the development of multi-component composite adsorbents and promotes the sustainable application of industrial by-products in environmental remediation.

## Figures and Tables

**Table 1:** Experimental conditions for Cd^2+^ and Pb^2+^ adsorption.

Experimental Projects	Variable Settings	Fixed Condition
One-way experiment	Dosage of RM: 0.01 g–0.12 g	[Cd^2+^] = 20 mg/L, [Pb^2+^] = 200 mg/L, pH = 6.0, Contact time = 1440 min
	pH: 3.0–7.0	[Cd^2+^] = 20 mg/L, [Pb^2+^] = 200 mg/L, Dosage of RM = 50 mg, Contact time = 1440 min
Kinetic experiment	Contact time: 15–1440 min	[Cd^2+^] = 20 mg/L, [Pb^2+^] = 200 mg/L, pH = 6.0, Dosage = 50 mg
Isothermal adsorption experiment	[Cd^2+^]: 5–80 mg/L, [Pb^2+^]: 50–800 mg/L	pH = 6.0, Dosage of RM = 50 mg, Contact time = 1440 min

**Table 2:** Physicochemical properties of biochars.

Biochar	pH	Yield (%)	Specific Area (m^2^/g)	Total Pore Volume (cm^3^/g)	Average Pore Diameter (nm)
BC	9.94	63.68	5.3207	0.0210	15.8109
RM	8.12	87.26	8.7773	0.0293	13.7793

**Table 3:** Kinetic model fitting parameters for Cd^2+^and Pb^2+^ adsorption by RM.

Ion	Pseudo-First-Order Kinetic			Pseudo-Second-Order Kinetic		
	K_1_	q_e_ (mg/g)	R^2^	K_2_	q_e_ (mg/g)	R^2^
Cd^2+^	5.0713	0.0661	0.2271	0.0041	6.1576	0.9971
Pb^2+^	22.305	0.0374	0.3361	0.0004	29.6209	0.9806

**Table 4:** Fitting parameters of the isothermal adsorption models for Cd^2+^ and Pb^2+^.

Ion	Langmuir Equation			Freundlich Equation		
	q_m_	K_L_	R^2^	K_F_	1/n	R^2^
Cd^2+^	12.13	0.0446	0.9557	1.1615	0.5051	0.9203
Pb^2+^	130.10	0.0020	0.8390	1.3540	0.6218	0.8511

## Data Availability

The original contributions presented in this study are included in the article. Further inquiries can be directed to the corresponding authors.
